# Cyanobacterial Toxin Degrading Bacteria: Who Are They?

**DOI:** 10.1155/2013/463894

**Published:** 2013-06-06

**Authors:** Konstantinos Ar. Kormas, Despoina S. Lymperopoulou

**Affiliations:** Department of Ichthyology & Aquatic Environment, School of Agricultural Sciences, University of Thessaly, 384 46 Volos, Greece

## Abstract

Cyanobacteria are ubiquitous in nature and are both beneficial and detrimental to humans. Benefits include being food supplements and producing bioactive compounds, like antimicrobial and anticancer substances, while their detrimental effects are evident by toxin production, causing major ecological problems at the ecosystem level. To date, there are several ways to degrade or transform these toxins by chemical methods, while the biodegradation of these compounds is understudied. In this paper, we present a meta-analysis of the currently available 16S rRNA and *mlrA* (microcystinase) genes diversity of isolates known to degrade cyanobacterial toxins. The available data revealed that these bacteria belong primarily to the Proteobacteria, with several strains from the sphingomonads, and one from each of the *Methylobacillus* and *Paucibacter* genera. Other strains belonged to the genera *Arthrobacter, Bacillus*, and *Lactobacillus*. By combining the ecological knowledge on the distribution, abundance, and ecophysiology of the bacteria that cooccur with toxic cyanobacterial blooms and newly developed molecular approaches, it is possible not only to discover more strains with cyanobacterial toxin degradation abilities, but also to reveal the genes associated with the degradation of these toxins.

## 1. Introduction

Cyanobacteria are some of the most “charismatic” microorganisms in the tree of life. Their ecological importance is widely recognised in the scientific world [1]. Among their remarkable features, though, there is a contradiction, at least for human interests. On one hand, they are considered of pronounced biotechnological interest as they produce several bioactive compounds through their metabolism, from biofuels and biopolymers to drugs [2–4]. On the other hand, they are capable of producing a wide range of nuisance secondary metabolites, that is, toxins (hereafter cyanotoxins). The toxicity of these compounds, which has been proved for a great variety of organisms [1], dictates for solutions to the problem caused by the accumulation of cyanotoxins in, mostly, freshwater water bodies all over the world. Although this issue of toxicity has been known for several decades now, there has been little effort towards biotechnological remedies, especially via degradation/transformation by microorganisms. This could be partially due to the notion that cyanotoxins are relatively recalcitrant to chemical degradation [5] and were thought as nonlabile for biodegradation as well. However, advances in molecular microbial ecology have elucidated that strains of the bacterial genera *Sphingomonas, Sphingosinicella, Arthrobacter, Brevibacterium, Rhodococcus, *and* Burkholderia* can degrade microcystins (MC) and nodularins in time scales from hours to days [6, 7]. Moreover, we are now aware of eukaryotic mechanisms of cyanotoxin elimination in animal tissues [8–10]. This slowly growing body of literature presents many opportunities for deeper investigations into cyanotoxins and their biodegradation.

 There is good evidence that toxic cyanobacterial water blooms favour the occurrence of specific members of the bacterioplankton [11]. These prokaryotes, bacteria and Archaea, are excellent candidates for having cyanotoxin-degrading properties. The satellite prokaryotic communities of cyanobacterial water blooms (e.g., [12, 13]) could either degrade and/or assimilate the toxin and their degradation products or could be inhibited by the toxins during the bloom (e.g., [14, 15]). Although there are several well-studied water bodies harbouring toxin-producing Cyanobacteria, their accompanying prokaryotic communities are considerably understudied (e.g., [12]). Another deficit in our current knowledge in cyanotoxin-degrading prokaryotes in such studies is that despite the fact that natural freshwaters can harbour diverse archaeal assemblages [16], practically nothing is known about the role of Archaea in cyanotoxin degradation. However, it has been shown recently that hydrogenotrophic methanogens are associated with *Microcystis* scum degradation [17], opening, thus, a new line of research for the investigation of the role of Archaea in MC and/or other cyanotoxin degradation.

### 1.1. Microcystins

Microcystins are the most widely distributed and known cyanotoxins. They consist of a group of cyclic heptapeptide hepatotoxins [cyclo-(D-Ala^1^-X^2^-D-MeAsp^3^-Z^4^-Adda^5^-D-Glu^6^-Mdha^7^)] produced by Cyanobacteria that belong to the genera *Microcystis, Anabaena, Nostoc,* and *Oscillatoria *(*Planktothrix*) [18, 19]. Two of these peptides (*X* and *Z*) are variable, resulting in more than 70 variants [20]. MC-LR, in which leucine (L) at position 2 and arginine (R) at position 4 are found, is the most toxic among MCs [21]. Conventional water treatment processes (chlorine, permanganate, chloride dioxide, ozone, and advanced oxidation methods) have been found inadequate for the removal of MCs [22]. Recently, more environmental friendly approaches have been applied to MC removal directly from natural eutrophic freshwater systems, involving plant material and minerals [23]. Whilst more advanced and efficient chemical techniques (granular activated carbon, powdered activated carbon, and membrane filtration) exist, they are considered too expensive to employ for the elimination of a contaminant whose presence in the water bodies is occasional and hard to predict [24]. Moreover, MCs' stable cyclic structure against physicochemical factors renders biodegradation inevitable, as the most sustainable, efficient, and realistic method for their removal [20]. Microbial degradation is the most important mechanism for the removal of MCs in the natural environment and is considered as an alternative water treatment strategy versus the physicochemical one [11].

**Figure 1:**
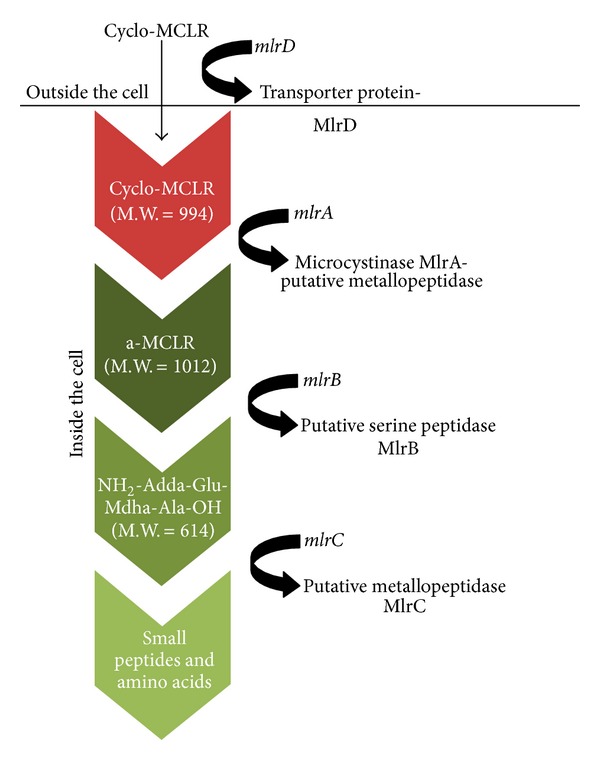
The degradative pathway of microcystin LR and the formation of intermediate (less toxic) products by *Sphingomonas* sp. strain ACM-3962. MW: molecular weight [25].

To date, only one biodegradation pathway for microcystin by *Sphingomonas* sp. strain ACM-3962 has been characterized [25]. The *mlr *gene cluster plays a crucial role in the sequential enzymatic hydrolyses of peptide bonds [21]. This cluster consists of four genes, that is, *mlrA, mlrB, mlrC*, and *mlrD,* and codes for at least three intracellular enzymes (Figure 1) [25].

The *mlrA* is the most important gene of this cluster because it encodes the enzyme that cleaves the Adda-Arg peptide bond in MC-LR and opens the cyclic structure [25]. The cyclic structure of MC is responsible for its stability against physicochemical and biological factors that contribute to inactivation or degradation, such as pH, temperature, sunlight, and other common proteases [26]. The initial hydrolysis mediated by the *mlrA *gene results in a substantial reduction in molecular toxicity, which is displayed by the 160-fold reduction of activity of the linear MC-LR towards protein phosphatase compared to that of the cyclic MC-LR. The *mlrA *gene sequence is very rare with no homologues found in the public databases, and therefore its functional characteristics are difficult to be assigned.

Biochemical characterization has shown that the *mlrA* gene encodes a metalloprotease [25], and its activation site consists of a typical to zinc metalloproteases motif, the HXXMECX [27]. It has been speculated that it might as well represent a new protease family with the function of cleaving smaller cyclic peptides [27]. The replacement of the R group by the A (alanine) group at MC-LA does not seem to affect the degradation process, suggesting that the presence of arginine may not be a critical site of cleavage [28]. In a recent study [29] the translation of the *mlrA* gene sequence and the alignment of the resulting amino acid sequence with other putative MlrA in the database revealed a zinc-bonding site and a transmembrane region, and the authors introduced the concept of a potential new protein family with unique functions. *mlrB *encodes a putative serine peptidase which degrades the linear (or acyclo) MC-LR to the tetrapeptide H-Adda-Glu-Mdha-Ala-OH, while MlrC exerts the action of a putative metallopeptidase and degrades the tetrapeptide to Adda or small amino acids [25]. The *mlrD *gene does not express hydrolytic activity and probably encodes the transporter protein that allows the uptake of MCs into the cell since it exhibits high sequence similarity to the PTR2 family of oligopeptide symporters [25].

 The MC degradation ability is not commonly present in the *Sphingomonas *genus but only in specific bacterial strains (Table 1) [27]. It has been suggested that the MC-degradation trait was acquired by gene transfer during the evolution of *Sphingomonas*, based on 16S rRNA gene analyses that relate phylogenetically non-MC-degrading bacteria closer to the MC-degrader strain Y2 [27]. Moreover, Manage et al. [30] managed to isolate bacteria that belonged to the Actinobacteria phylum (*Arthrobacter *sp.,* Brevibacterium *sp., and *Rhodococcus *sp.) capable of degrading MC-LR. The *mlr *gene cluster was not detected, but this is the first report on bacteria other than Sphingomonadaceae with the ability to degrade MCs. Hu et al. [51] also isolated a *Methylobacillus *sp. strain from a cyanobacterial sludge with the ability to completely degrade both MC-LR and MC-RR. To date, 20 different MC-degrading bacteria have been isolated from rivers, lakes, and biological filters, but without simultaneous detection of the *mlr* gene cluster, which is considered to encode the hydrolytic proteins involved in the initial steps of MC-degradation [41]. It seems that the presence of microbial populations capable of degrading MCs and other peptides is promoted by the prevalence of MC-containing blooms [20]. Therefore, a greater diversity of bacterial genera might be able to degrade MCs with as yet to be characterized degradation mechanisms.

### 1.2. Nodularins

Nodularins are produced by *Nodularia spumigena *and have been detected mostly in brackish habitats [28, and references therein]. Their structure is similar to that of MCs with the difference that they consist of a pentapeptide [cyclo-(D-MeAsp^1^-L-Arg^2^-Adda^3^-D-Glu^4^-Mdhb^5^)] [20]. Consequently, some bacteria which are capable of degrading MCs are also able to degrade NODs (Table 1), possibly due to the similar mode of action of MlrA that cleaves hydrolytically their cyclic structure at the Adda-Arg peptide bond [28].

### 1.3. Cylindrospermopsin

 Cylindrospermopsin (CYN) is a group of alkaloid cytotoxins which are produced by *Cylindrospermopsis raciborskii*, *Umezakia natans*, *Anabaena bergii*, *Anabaena lapponica*, *Aphanizomenon ovalisporum*, *Aphanizomenon flosaquae*, *Raphidiopsis curvata*, and *Lyngbya wollei*. Three studies until now have demonstrated that CYN can be biodegraded in water habitats [55, 56]. Smith et al. [55] found that a linear relation between the biodegradation rate and the initial concentration of CYN could be established, while Mohamed and Alamri [56] reported the biodegradation of CYN by a *Bacillus* strain (AMRI-03) isolated from a cyanobacterial bloom, with rapidly occurring degradation rates, highly dependent on the initial CYN concentration. However, no defined metabolic pathway has been elucidated to date.

### 1.4. Saxitoxins

Saxitoxins form a group of alkaloid neurotoxins that can be produced by dinoflagellates and many different cyanobacterial genera, including *Anabaena, Raphidiopsis, Lyngbya, Planktothrix (Oscillatoria),* and *Cylindrospermopsis* [18, 57]. While there are many studies that report the biotransformation of saxitoxin variants to more toxic ones [58, 59], there is scarcity of literature regarding its biodegradation. Donovan et al. [60] demonstrated an overall reduction of saxitoxin mixture to 90% by seven unidentified but potential saxitoxin-degrading bacteria that had been isolated from the digestive tracts of blue mussels.

### 1.5. Anatoxins

 Anatoxin-a is a low molecular weight neurotoxic alkaloid that is produced by cyanobacteria belonging to the genera *Anabaena*, *Aphanizomenon*, *Microcystis*, *Planktothrix*, *Raphidiopsis*, *Arthrospira*, *Cylindrospermum*, *Phormidium*, *Nostoc,* and *Oscillatoria* [61]. There is limited information on the biodegradation of anatoxin-a. A *Pseudomonas *sp. isolate was reportedly able to degrade anatoxin-a [62], while Rapala et al. [63] also reported a significant (22–48%) reduction of anatoxin-a in sediments, but no further information on bacteria able to degrade or enzymatic pathways and corresponding genes is available.

### 1.6. *β*-N-Methylamino-L-Alanine (BMAA)

BMAA is a highly reactive nonessential amino acid which can be found either free or protein-bound. It is associated with amyotrophic lateral sclerosis and Parkinson dementia complex (ALS/PDC) [64]. Although BMAA is the most recent cyanotoxin discovered to date [65], it is produced from members from all major cyanobacterial groups [66] and for this reason it is believed to be widespread in freshwater systems. It has been demonstrated that it accumulates in higher trophic levels, including species that are consumed by humans [64]. As airborne algae and Cyanobacteria [67] have started to attract the scientific interest in a more focused way [68], it has been proposed recently that it can be dispersed via aerosolization of Cyanobacteria containing this toxin [69]. To date, no published data exist on the biodegradation on BMAA.

### 1.7. Nontoxic Nuisance Cyanobacterial Compounds

 Apart from the cyanotoxins, the water quality problems in freshwater bodies are also related to the organoleptic traits of water which are easily perceived by consumers due to the altered and usually unpleasant taste and odor. 2-Methylisoborneol (MIB) and geosmin, two cyclic aliphatic tertiary alcohols, are the main responsible compounds that impart such problematic characteristics in waters (earthy/camphorous odour), even though they both do not pose any known hazard for the human health [28]. MIB and geosmin can be produced by a range of Cyanobacteria including *Anabaena*, *Aphanizomenon*, *Geitlerinema*, *Symploca*, *Planktothrix* (*Oscillatoria*), *Phormidium*, *Nostoc*, *Pseudanabaena*, and *Lyngbya* [70]. There are several studies which elucidate the ability of some bacteria to biodegrade both compounds [71–74], but no definite metabolic pathway is known. The biodegradation reactions of both compounds seem to be mediated by monooxygenase enzymes in a way similar to the biological Baeyer-Villiger reaction that takes place during the biodegradation of camphor, a bicyclic ketone [75]. This is due to the similarity of their structure to alicyclic alcohols and ketones [75, 76]. The isolation and cloning of the *cam *operon from a camphor-degrading strain of *Pseudomonas putida *confirmed this hypothesis [77], while camphor enrichment of a camphor-degrading bacterial consortium has led to the isolation of all three available secondary metabolites of MIB [74]. Similarly, the biodegradation of geosmin has been reported by cyclohexanol-degrading strains of *Nocardia *and *Acinetobacter*, equivalently to the Baeyer-Villiger reaction [75], while Eaton and Sandusky [74] demonstrated geosmin biodegradation by two terpene-degrading bacteria, *Rhodococcus wratislaviensis* DLC-cam and *Pseudomonas* sp. SBR3-tpnb, but only after their induction with either camphor or terpene.

In this paper, we review the available literature on the molecular diversity of bacterial strains which possess some kind of cyanotoxin-degrading feature or, at least, are naturally associated with toxin-producing Cyanobacteria based on 16S rRNA and *mlrA* gene sequence diversity. We aimed to depict the major taxa that include such strains as a first step in a focused approach for the isolation and biotechnological development of microorganisms that can degrade cyanotoxins in natural and engineered aquatic systems.

## 2. Materials and Methods

16S rRNA gene sequences of bacterial isolates that either carry the *mlrA* gene or grow on/degrade MCs or both were retrieved from GenBank, and phylogenetic trees were constructed using the neighbor-joining algorithm based on distances calculated by the Jukes-Cantor model and implemented in the MEGA5 software [78]. Bootstrapping was performed with 1,000 replicates to assign confidence levels to the tree topology. The sequences were screened for chimeras using the Bellerophon program at http://comp-bio.anu.edu.au/bellerophon/bellerophon.pl/. No putative chimerical sequences were detected.

The search of *mlrA* sequences in the GeneBank database proved a not so straight forward procedure, as the search by using “*mlrA*” gives ambiguous results, with some of them being totally irrelevant to the degradation gene. An initial search of the “*mlrA*” gene turned back several hundreds of sequences. Most of these sequences are associated with *Escherichia coli* and representatives of the family of Enterobacteriaceae that correspond to genes unrelated to the degradation of microcystins. The majority of these genes are related to transcriptional regulators such as the HTH-type transcriptional regulator, the ABC-transporter, acetyl-xylan esterases, the McrR-like regulator A, and the merR family. Filtering of these results by excluding all *E. coli* and Enterobacteriaceae bacteria (e.g., *mlrA* [All Fields] NOT “Escherichia” NOT “Shigella” NOT “Klebsiella” NOT “Enterobacter” NOT “Salmonella” NOT “Cronobacter” NOT “Erwinia” NOT “Citrobacter” NOT “Pantoea”) shrinks the results to a few tenths of sequences that still include some irrelevant sequences similar to those described above. After this stage, manual fishing of the *mlrA* sequences was necessary. Two sequences of uncultured clones (FJ438526 and FJ438527) were excluded due to their short length (120 bp). Four more sequences of uncultured clones were excluded (AB600656, AB600658, AB600663, and AB600668) due to the high number of potential but short amino acid (1–157 aa) ORFs into which they could be translated. The MlrA sequences we used consisted of 184 or more amino acids.

The amino-acid sequences of the *mlrA *gene-carrying bacterial strains were retrieved from the GenBank (http://www.ncbi.nlm.nih.gov/nuccore), and their phylogenetic relationship was inferred using the neighbor-joining method. The evolutionary distances were computed using the Poisson correction method. Bootstrapping was performed with 1,000 replicates. *Sphingopyxis* sp. C-1′s amino-acid sequence was not available in the database and was translated by the nucleotide sequence with the EMBOSS (Sixpack)_program (http://www.ebi.ac.uk/Tools/st/). There were a total of 157 informative positions in the final dataset.

## 3. Results and Discussion

**Figure 2:**
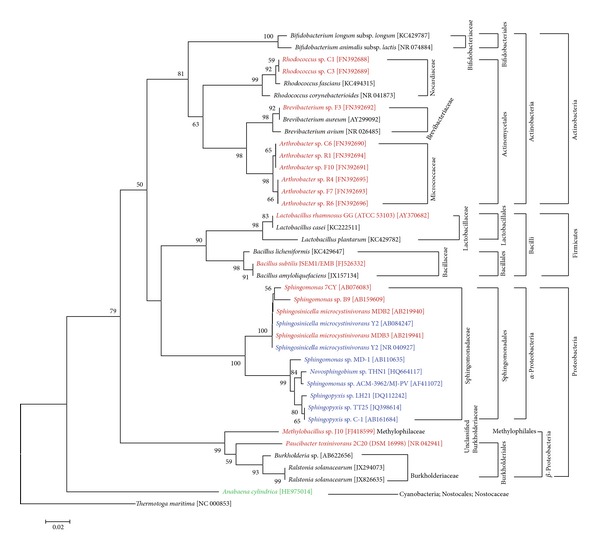
Phylogenetic tree of the 16S rRNA gene sequences of isolates that either carry the *mlrA *gene (in green) or degrade MC (in red) or both (in blue), based on the neighbour-joining method and a Jukes-Cantor distance matrix. One thousand bootstrap analyses were conducted, and percentages greater than 50% are indicated at nodes. The numbers in brackets are GenBank accession numbers. *Thermotoga maritima *was used as an outgroup. Scale bar represents 2% estimated distance.

**Figure 3:**
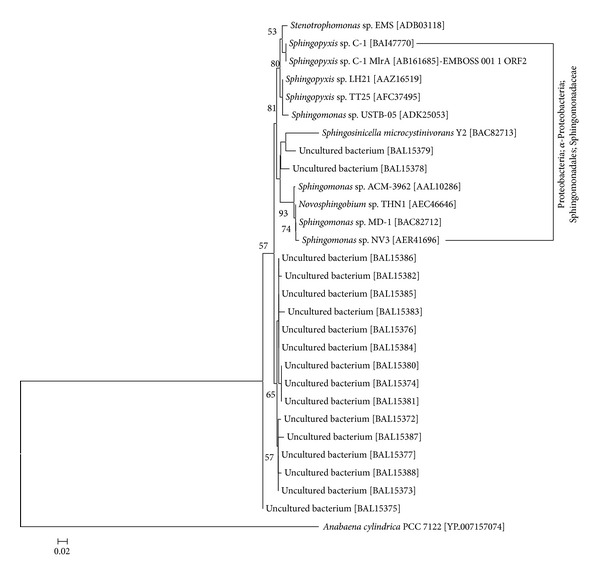
Phylogenetic tree MlrA amino acid sequences retrieved from the GenBank database, based on the neighbor-joining method. The evolutionary distances were computed using the Poisson correction method. One thousand bootstrap analyses were conducted, and percentages greater than 50% are indicated at nodes. The numbers in brackets are GenBank accession numbers. Scale bar represents 2% estimated distance.

 Our intention was to identify ways to enhance the development of biotechnological tools for biodegradation of cyanotoxins by reviewing the current literature and mining databases for possible new prokaryotes of interest. The bulk of the existing data deal with microcystins. Our analysis revealed that the ubiquitous *α*- and *β*-Proteobacteria and Actinobacteria include the majority of potential cyanotoxin-degrading bacteria (Figure 2). Based on their phylogenetic taxonomy, the few recently found strains capable of degrading microcystin or carrying the *mlrA* gene were closely affiliated to known species or genera (Figure 2). The analysis of the MlrA protein diversity (Figure 3) showed that all the available sequences also belong to closely related members of the Sphingomonadaceae (*α*-Proteobacteria), but there are also several strains that cannot be assigned to known bacterial taxa.

The majority of the taxa with cyanotoxin degradation capability are known to have multiple other biodegradation traits. Strains of the genus *Rhodococcus* are known for degrading xenobiotics in various aquatic and terrestrial habitats and also for producing surfactants [79]. The strains that belonged to the Sphingomonadaceae family were affiliated with the genera *Sphingosinicella*, *Sphingopyxis*, *Sphingomonas,* and *Novosphingobium*. The sphingomonads are not new to biotechnological applications. They are involved with novel catalyses, bioremediation, fossil fuel desulfurization, novel enzymes, biotin, and polysaccharide production. Their ability to degrade hazardous organic compounds, for example, PCBs, creosote, pentachlorophenol, herbicides, and the conversion of commonly occurring organics to novel or specialty chemicals, is a key feature for the group, especially in contaminated soils and sediments [80, and references therein]. Despite their biotechnological significance and their widespread distribution, their ecology is insufficiently studied. This could be due to their ability to utilize a wide array of organic-often refractory substances, their metabolic diversity, and their ability to grow in oligotrophic conditions, with the latter being more obvious in the marine environment. These features also hinder their successful cultivation. The metabolic diversity of this group is indicated by the lack of a culture medium specific for sphingomonads, leaving the molecular approaches more appropriate for their study. They are more frequently found in freshwater habitats like rivers, ponds, lakes, and groundwater, but the majority of the available strains have been isolated from contaminated to heavily contaminated sites [80, and references therein]. Since these strains have been found to carry the *mlrA* gene and/or grow on microcystin, it is plausible to consider them as some of the most active—and promising for future applications—players in the degradation of microcystin.

Microcystins and nodularins' biosynthesis is also carried out by nonribosomal peptide synthetases (NRPS) and type I polyketide synthases (PKS-I) [81]. These extraordinary enzymes (along with their products) are evolved rapidly through multiple mechanisms [82]. On the other hand, the ability of the cooccurring bacterioplankton in cyanobacterial water blooms to degrade/assimilate toxins (e.g., [14, 15]) along with the metabolic diversity, but insufficiently studied ecology, of bacteria such as the sphingomonads [80, and references therein] is well documented. These two adversary forces might have an important ecological function and might be able to drive the coevolution of the NRPS/PKS-I enzymes and the cyanotoxin biodegradation pathways in a water bloom, leading to a “race of arms” against cyanotoxins. Moreover, the retrieval of bacteria such as Actinobacteria that lack the known *mlr* gene cluster [30] implies the existence of more genes, and thus pathways, associated with the degradation of these toxins.

The genus *Arthrobacter* (Figure 2), originating mostly from soil, is known in biotechnology mostly because of its nutritional versatility of growing on a wide array of organic compounds, including herbicides, pesticides, *n*-alkanes, aromatic compounds, and lower alcohols [83, and references therein], its potential for bioremediating their heavy metals (Cd, Co, Cu, Ni, and Pb) [79], and also for the production of surfactants, glutamic acid, *α*-ketoglutaric acid, and riboflavin [79, 83]. The ability of *Arthrobacter* to degrade microcystins renders this genus an important one in future biotechnological quests. Similar features are also found in *Brevibacterium* spp. (Figure 2), a genus that is taxonomically and physiologically similar to *Arthrobacter,* and until recently, frequently confused with it [81].

*Bacillus* spp. have been one of the pillars of biotechnology for several decades now. Although they possess numerous biodegradation competences, to date only one isolate (Figure 2) seems to be associated with the microcystin degradation. This by no means diminishes the biotechnological value of this genus; the reason for the underrepresentation of *Bacillus* spp. is possibly due to the fact that the Firmicutes is a minor phylum in freshwater habitats [84] where most toxic cyanobacterial blooms occur.

Apart from the above-mentioned strains with already well-recognised biodegradation potential, the *Methylobacillus* sp. and *Paucibacter* sp. (Figure 2) can be considered important emerging genera of microcystin degraders. This suggests that with ongoing research and the application of appropriate methodology new taxa of degraders can be revealed.

It has been shown that freshwater harbors a vast and distinguishable bacterial diversity from other aquatic habitats [84, 85]. However, our meta-analysis showed that, to date, only a few bacterial species bear the *mlrA* gene, a proxy for microcystin degradation potential. Moreover, several of these bacteria are not abundant in the ecosystems where microcystins and other cyanotoxins are found. This could partially explain our perceived concept of the nonlabile nature of microcystins and possibly of other cyanotoxins. However, it could also be related to the methodological approaches used so far to isolate such degraders. The most common practice involves culture-dependent approaches with the toxin being the sole carbon and/or energy source (e.g., [39, 48]). We believe that novel/recent molecular methodologies provide valuable complementary information because they overcome culturing limitations, and in silico approaches are not dependent on metabolic traits of the taxa containing genes for microcystin degradation.

Nowadays there are several omics methodologies that can be tailored to specific scientific quests. Future investigations targeting microorganisms with cyanotoxin degradation could include (a) genomic analysis of the available strains, like the case of *Sphingosinicella microcystinivorans* [39]; gene mining of such genomes could depict the relevant degradation pathways; (b) metagenomic libraries of habitats where the sphingomonads are either abundant or with bacterial communities occurring in close association with toxic cyanobacterial blooms and followed by metatranscriptomics libraries of the same samples where the MlrA and other related enzymes have been proved to be transcribed (c) single-cell genomics (SCG). SCG is of particular interest to biotechnology and bioprospecting due to its ability to attribute specific—often novel—biochemical pathways to specific cells/species [31]. Recently, a SSG study revealed for the first time a great extent of the metabolic potential of a ubiquitous freshwater actinobacterial species [86]. In the case of cyanotoxin degraders, for example, it would be feasible to assign the biodegradation pathway of cyanotoxins to specific bacteria (i.e., single-cells) from any toxic cyanobacterial water bloom, regardless of their cultivability. The application of such approaches, along with the development of standardized and easy-to-use analytical methods for the measurement of multiple cyanotoxins from the same sample, could speed progress towards the standardized usage of specific cyanotoxin degraders. Finally, special attention should be paid to other toxins than the microcystins, as some of these are, at least, of equal potency, distribution, and persistence in the environment.

## Figures and Tables

**Table 1:** Isolated microcystin degrading Bacteria.

Bacteria	Source	16S rRNA gene GenBank accession number	Degradable analogues	MC-degrading genes	Degradation of other toxins	References
Actinobacteria						
*Arthrobacter* sp.	Surface water	FN392690, FN392691, FN392693, FN392694, FN392695, FN392696	MCLR, MCRR, MCLF	—	NOD	[30]
*Brevibacterium* sp.	Surface water	FN392692	MCLR, MCRR, MCLF	—	NOD	[30]
*Rhodococcus* sp.	Surface water	FN392688, FN392689	MCLR, MCRR, MCLF	—	NOD	[30]
*Microbacterium *sp.	Lake water	—	MCLR	Unknown		[31]
*Bifidobacterium lactis *Bb12 and 420	Probiotic strains	—	MC-LR, MC-RR, MC-YR, MC-LF, MC-LY, MC-LW	Unknown	CYN	[32, 33]
*Bifidobacterium longum *46	Probiotic strains	—	MC-LR, MC-RR, MC-YR, MC-LF, MC-LY, MC-LW	Unknown	CYN	[32, 33]
Firmicutes						
*Bacillus *sp. EMB/JSEM1	Artificial media	FJ526332	MCLR, MCRR	*mlrA *		[34]
*Lactobacillus rhamnosus *GG and LC-705	Probiotic strains	AY370682	MC-LR, MC-RR, MC-YR, MC-LF, MC-LY, MC-LW	Unknown	CYN	[32, 33]
*α*-Proteobacteria						
*Sphingomonas* sp. ACM-3962 or MJ-PV	Surface water	AF411072	MCLR, MCRR	*mlrA* *mlrB* *mlrC* *mlrD*		[35]
*Sphingomonas* sp. 7CY	Lake water	AB076083		Unknown		[36]
*Sphingomonas* sp. B9	Lake water	AB159609	MCLR, MCRR, NOD	Unknown	NOD	
*Sphingomonas* sp. CBA4		AY920497		Unknown		[37]
*Sphingomonas* sp. MD-1	Lake water	AB110635	MCLR, MCRR, MCYR	*mlrA *		[27, 38]
*Sphingomonas* sp. MDB2 (*Sphingosinicella* sp.)	Lake water	AB219940	MCLR, MCRR, MCYR	Unknown		[39]
*Sphingomonas* sp. MDB3 (*Sphingosinicella* sp.)	Lake water	AB219941	MCLR, MCRR, MCYR	Unknown		[39]
*Sphingomonas* sp. Y2 (*Sphingosinicella microcystinivorans*)	Lake water	NR_040927/AB084247	MCLR, MCRR, MCYR	*mlrA *		[40]
*Sphingomonas stygia *	Lake water	—	MC-LR, MC-RR, MC-YR	Unknown		[38]
*Sphingopyxis* sp. LH21	Biological sand filter	DQ112242	MCLR, MCLA	*mlrA* *mlrB* *mlrC* *mlrD *		[41–43]
*Sphingopyxis* sp. USTB-05		—	MCLR, MCRR, MCYR, MCLA	*mlrA *		[44–46]
*Sphingopyxis *sp. TT25	Reservoir water	JQ398614	MCLR, MCRR, MCYR, MCLA	*mlrA *		[28]
*Sphingomonas *sp. NV3	Lake water	JN256930	Unknown	*mlrA *		Unpublished
*Novosphingobium *sp. THN1	Lake water	HQ664117	MCLR	*mlrA* *mlrB* *mlrC* *mlrD *		[47]
*Sphingopyxis* sp. C-1	Water bloom	AB161684	MCLR	*mlrA *		[48]
*Rhizobium gallicum *	Lake water	—	MCLR	Unknown		[49]
*β*-Proteobacteria						
*Burkholderia* sp.	Surface water	DQ459360	MCLR	Unknown		[50]
*Methylobacillus* sp. J10	Sludge from Cyanobacteria salvaged yard	FJ418599	MCLR, MCRR	—		[51]
*Paucibacter toxinivorans *	Lake sediment	NR_042941	MCLR, MCYR, NOD	—		[52]
*Ralstonia solanacearum *		—		—		[53]
*γ*-Proteobacteria		—		—		
*Morganella morganii *	Lake water active anthracite biofilter	—	MCLR	—	NOD	[24]
*Pseudomonas aeruginosa *	Lake water	—	MCLR	—		[54]
*Stenotrophomonas* sp. EMS	Lake water	—	MCLR, MCRR	*mlrA *		[29]

CYN: cylindrospermopsin, NOD: nodularin, MC: microcystin, A: alanine, F: phenylalanine, L: leucine, R: arginine, Y: tyrosine, W: tryptophan, *mlr*: mycrocystinase gene.
